# Context‐dependency in carnivore co‐occurrence across a multi‐use conservation landscape

**DOI:** 10.1002/ece3.9239

**Published:** 2022-08-29

**Authors:** Gonçalo Curveira‐Santos, Laura Gigliotti, Chris Sutherland, Daniela Rato, Margarida Santos‐Reis, Lourens H. Swanepoel

**Affiliations:** ^1^ Centre for Ecology, Evolution and Environmental Changes (cE3c), Faculdade de Ciências Universidade de Lisboa Lisbon Portugal; ^2^ Department of Environmental Science, Policy, and Management University of California Berkeley Berkeley CA USA; ^3^ Centre for Research into Ecological and Environmental Modelling University of St Andrews St Andrews UK; ^4^ Department of Zoology, School of Mathematical & Natural Sciences University of Venda Thohoyandou South Africa; ^5^ African Institute for Conservation Ecology Levubu South Africa

**Keywords:** camera trap, conservation management, co‐occupancy, interspecific interactions, temporal overlap

## Abstract

Carnivore intraguild dynamics depend on a complex interplay of environmental affinities and interspecific interactions. Context‐dependency is commonly expected with varying suites of interacting species and environmental conditions but seldom empirically described. In South Africa, decentralized approaches to conservation and the resulting multi‐tenure conservation landscapes have markedly altered the environmental stage that shapes the structure of local carnivore assemblages. We explored assemblage‐wide patterns of carnivore spatial (residual occupancy probability) and temporal (diel activity overlap) co‐occurrence across three adjacent wildlife‐oriented management contexts—a provincial protected area, a private ecotourism reserve, and commercial game ranches. We found that carnivores were generally distributed independently across space, but existing spatial dependencies were context‐specific. Spatial overlap was most common in the protected area, where species occur at higher relative abundances, and in game ranches, where predator persecution presumably narrows the scope for spatial asymmetries. In the private reserve, spatial co‐occurrence patterns were more heterogeneous but did not follow a dominance hierarchy associated with higher apex predator densities. Pair‐specific variability suggests that subordinate carnivores may alternate between pre‐emptive behavioral strategies and fine‐scale co‐occurrence with dominant competitors. Consistency in species‐pairs diel activity asynchrony suggested that temporal overlap patterns in our study areas mostly depend on species' endogenous clock rather than the local context. Collectively, our research highlights the complexity and context‐dependency of guild‐level implications of current management and conservation paradigms; specifically, the unheeded potential for interventions to influence the local network of carnivore interactions with unknown population‐level and cascading effects.

## Graphical abstract

We explored assemblage‐wide patterns of carnivore spatial (residual occupancy probability) and temporal (diel activity overlap) co‐occurrence across three adjacent wildlife‐oriented management contexts in South Africa ‐ a provincial protected area, a private ecotourism reserve, and commercial game ranches. We found that carnivores were generally distributed independently across space, but existing spatial dependencies were context‐specific. Our research highlights the complexity of guild‐level implications of current management and conservation paradigms." cd_value_code="text

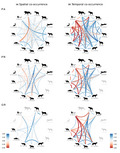

## 1 INTRODUCTION

How species' resource use affinities and interspecific interactions act to structure animal communities is a central tenet of coexistence theory (Macarthur & Levins, 1967; Schoener, 1974). Research on spatial and temporal species' co‐occurrence patterns (i.e., relative spatial and diel activity distributions) within mammalian carnivore guilds has assumed particular relevance (e.g., Davis et al., 2018). This is largely due to carnivores' propensity for agonistic interactions (Caro & Stoner, 2003; Donadio & Buskirk, 2006; Ritchie & Johnson, 2009) and their influence over ecosystem processes and functioning (Estes et al., 2011; Prugh et al., 2009; Ripple et al., 2014). Importantly, ongoing global declines of apex predators and shifts toward mesopredator‐dominated systems (Hoeks et al., 2020) have motivated increasing calls for a more comprehensive understanding of predator community structure and its inclusion in conservation and restoration plans (Jachowski et al., 2020; Ritchie et al., 2012; Ritchie & Johnson, 2009; Svenning et al., 2016).

Fine‐scale coexistence of sympatric carnivores species is mediated by a complex interplay of interspecific interactions beyond individual resource preferences and species' fundamental niches (Rosenzweig, 1966). Behavioral adjustments associated with interspecific interactions include changes in space use and circadian activity, to avoid confrontation with dominant species and/or partition the use of common resources (Karanth et al., 2017; Mills et al., 2019; Monterroso et al., 2014, 2020; Robinson et al., 2014). Outcomes of antagonistic interactions may induce suppression‐driven cascades whereby apex predators limit large‐bodied mesopredators, indirectly benefiting smaller carnivores (Levi & Wilmers, 2012). Conversely, facilitative interactions, such as carrion provisioning by large carnivore hunts, may promote carnivore co‐occurrence and even enhance suppression at the population level (Prugh & Sivy, 2020; Sivy et al., 2017). When interspecific aggression and competition are not the primary drivers, co‐occurrence in space and time can result from trait similarities and common environmental and resource affinities or stressors (i.e., habitat filtering; Rich et al., 2017). The interplay of co‐occurrence patterns becomes particularly diffuse in species‐rich carnivore assemblages, where heterogeneity in species' behavior, morphology, and phylogeny influences the nature and strength of interspecific interactions at local scales.

A growing body of literature has helped elucidate the complexity of carnivore community structure and intraguild interactions. Less attention, however, has been given to underlying context‐dependency, which is generally assumed but often poorly described (Bar‐Massada & Belmaker, 2017; Chamberlain et al., 2014). Differences in community composition and abundance of local carnivore species, as well as diversity, availability, or spatial structuring of resources, can underly context‐specific carnivore spatiotemporal dependencies (Karanth et al., 2017). Human‐caused disturbances are often the dominant driver in forming ecological context, by modifying the landscapes and communities that predators interact with and within, albeit with varying effects in direction and magnitude (reviewed in Sévêque et al., 2020). Human influence often has broad and heterogeneous effects by, for instance, reducing risk‐free and undisturbed space, inducing nocturnality, providing reduced or surplus resources (e.g., Curveira‐Santos et al., 2017; Mills & Harris, 2020). Hence, anthropogenic influence may artificially narrow or dilute spatiotemporal scopes for carnivore interactions, thus altering the landscape of coexistence (Oriol‐cotterill et al., 2015). Moreover, anthropic influence on apex predators is particularly accentuated and well‐described, ranging from harmful interventions (e.g., direct persecution; Treves & Karanth, 2003) to directed conservation initiatives (Mossaz et al., 2015; Sergio et al., 2008), influencing these species' ability to regulate guild and ecosystem dynamics (Dorresteijn et al., 2015; Haswell et al., 2015; Kuijper et al., 2016). Carnivore communities of similar composition may thus exhibit fundamentally different spatiotemporal structures across ecological contexts and, subsequently, varying interaction dynamics.

South Africa's diverse carnivore assemblages and the complexity of the highly variable local conservation landscape are a pertinent model system to explore context‐specific carnivore co‐occurrence patterns. Intraguild killing and interference exploitative competition are pervasive among African carnivores (Caro & Stoner, 2003). Moreover, empirical evidence supports suppressive effects by the dominant apex predator, the African lion (*Panthera leo*), and other large carnivores, while the wide range of carnivore species' body sizes presupposes potential suppression‐based cascades (Levi & Wilmers, 2012). Carnivore sympatry in South African landscapes is thus expected to arise via a complex interplay of behavioral mechanisms and co‐occurrence patterns (Durant, 1998; Hayward & Slotow, 2009; Mills et al., 2019; Ramesh et al., 2017).

Notably, however, widespread human encroachment, wildlife fencing (Packer et al., 2013), and decentralized approaches to conservation (Pitman et al., 2017) have markedly altered the stage upon which carnivore interactions take place. Private solutions to natural resource management have increasingly complemented the formal network of protected areas following changes in wildlife ownership rights, which prompted a large‐scale land‐use shift from livestock farming to game ranching (ecotourism and/or hunting; Pitman et al., 2017), giving rise to intricate multi‐tenure conservation landscapes (Curveira‐Santos, Sutherland, Santos‐Reis, & Swanepoel, 2021a; Di Minin et al., 2013). While large carnivores have been widely reintroduced into small and fenced reserves and often maintained at high densities (Mossaz et al., 2015), human presence and persecution of free‐ranging species on game ranchland is common practice (Lindsey et al., 2008). Changes in guild composition are accompanied by varying levels of human disturbance and its influence on resource availability across multiple land uses. Together, these can deeply shape the structure of carnivore assemblages (Curveira‐Santos, Sutherland, Tenan, et al., 2021b; Schuette et al., 2013) and potentially alter the underlying network of species interactions and community regulation pathways (Dorresteijn et al., 2015). Nonetheless, context‐dependent changes in carnivore assemblages have received little attention in carnivore‐rich regions of southern Africa, where context‐dependency is likely to have profound effects.

Here, we link heterogeneity in carnivore co‐occurrence patterns to variation in conservation management models implemented in South Africa. We evaluate spatial (residual occupancy correlation) and temporal (diel activity overlap) carnivore co‐occurrence patterns across three adjacent wildlife‐oriented management contexts, spanning a 109‐year‐old provincial protected area (“conservation reference”), a private ecotourism game reserve, and a mosaic of commercial game ranches (see Curveira‐Santos, Sutherland, Santos‐Reis, & Swanepoel, 2021a). Specifically, we aim to assess context‐dependent variation in the prevalence of positive and negative spatial and temporal associations, at both the assemblage and species‐pair levels. Additionally, we ask whether drivers of carnivore co‐occurrence vary across areas following main theoretical interaction pathways; namely “competitive exclusion” and “limiting similarity” principles (Macarthur & Levins, 1967). We hypothesized that carnivore spatial and temporal co‐occurrence would differ among management contexts. We predicted that: (1) spatial and temporal co‐occurrence would be higher in the formally protected area because of higher species abundances and more stable community composition (Curveira‐Santos, Sutherland, Santos‐Reis, & Swanepoel, 2021a); (2) spatial and temporal co‐occurrence would follow a dominance hierarchy in the private reserve because of higher apex predator (lion) densities (Ritchie & Johnson, 2009); (3) spatial and temporal co‐occurrence would not vary based on a dominance hierarchy and would overall be positive in game ranches because of a depauperate large carnivore guild and more limited disturbance‐free spaces due to predator persecution (Sévêque et al., 2020); and (4) regardless of management context, spatial and temporal co‐occurrence would be inversely related according to niche partitioning theory (Schoener, 1974).

## 2 METHODS

### 2.1 Management contexts and carnivore surveys

**FIGURE 1:**
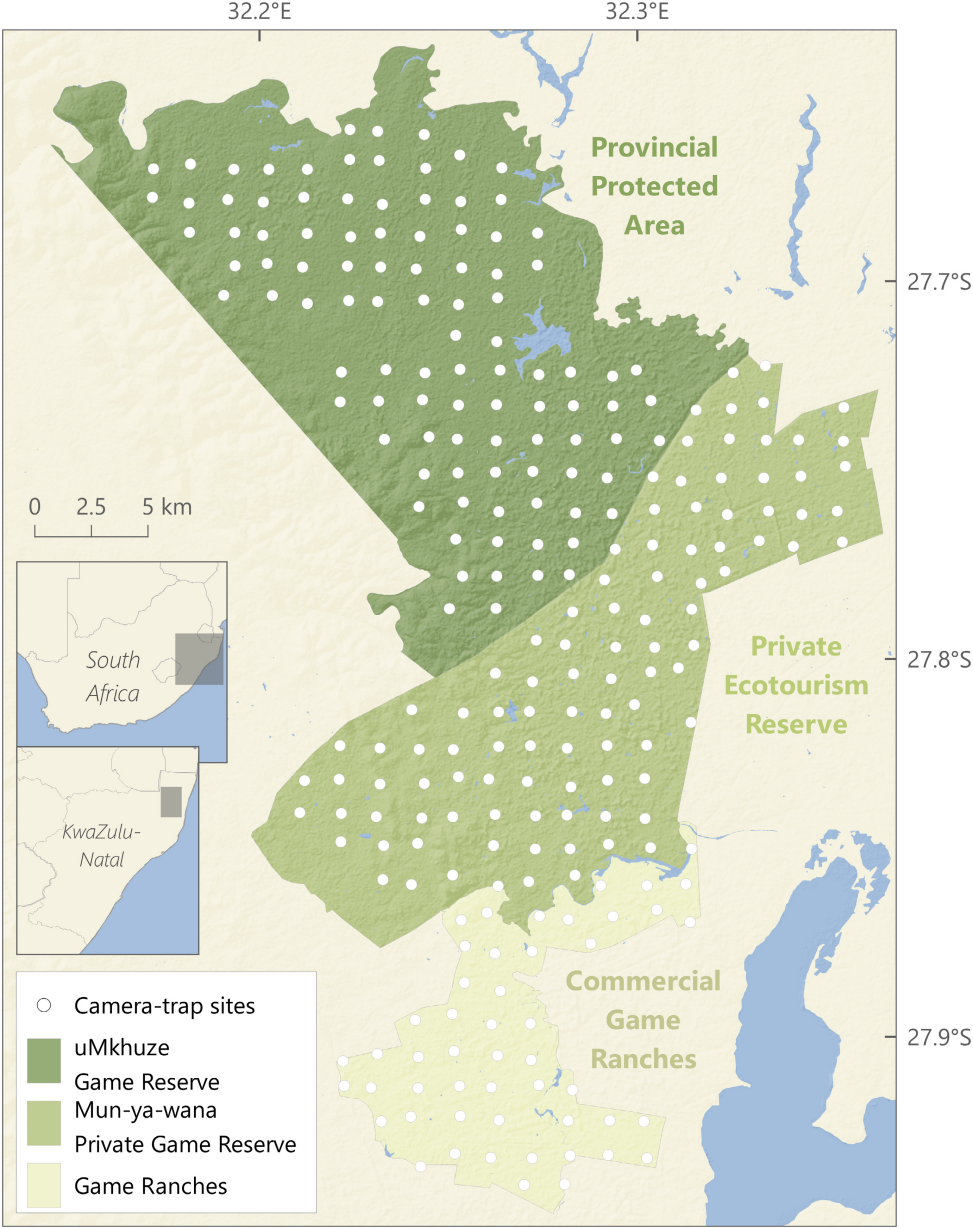
Camera‐trapping surveys across three adjacent wildlife‐oriented management contexts (provincial protected area, private ecotourism reserve, and commercial game ranches) in the Maputaland region of northern KwaZulu‐Natal, South Africa

We used data from carnivore camera‐trap surveys carried out in the uMkhuze—Mun‐Ya‐Wana complex and surrounding game ranchland, in the Maputaland‐Pondoland‐Albany Biodiversity Hotspot, NE KwaZulu‐Natal, South Africa (Figure 1). The three target areas form a spatial continuum with similar prevailing vegetation (savanna broad‐leaf woodland varieties [*Vachellia* and *Terminalia*] interspersed with open and semi‐open wooded‐grasslands) but contrasting management priorities and varying carnivore assemblages (Curveira‐Santos, Sutherland, Santos‐Reis, & Swanepoel, 2021a). uMkhuze Game Reserve (PA) is a provincially managed protected area established in 1912 as part of iSimangaliso Wetland Park, a UNESCO World Heritage Site. The Mun‐ya‐wana Private Game Reserve (PR) is a private wildlife reserve composed of multiple properties with no internal fencing, managed primarily for ecotourism since 1991. Both reserves hold a near‐complete suite of large carnivores, although African wild dogs (*Lycaon pictus*) only occasionally visit the PR. The mosaic of commercial game ranches (GR) to the south is dedicated to the production of wild ungulate species, and occasionally domestic cattle, with large expanses of natural habitat and low human densities. Leopards (*Panthera pardus*) and spotted hyenas (*Crocuta Crocuta*) are the only large carnivores that are observed in GR, where both experience widespread persecution (Balme et al., 2010). The species composition of medium‐ (5–20 kg) and small‐sized (<5 kg) carnivores is similar across all areas but occupancy rates are lower in the PR and GR compared with the PA. See Curveira‐Santos, Sutherland, Santos‐Reis, and Swanepoel (2021a) for a detailed assessment of variation in carnivore assemblage composition and species occupancy across the focal landscape.

The three study areas were sampled during the late dry season (Aug–Nov) using a uniformly spaced camera‐trapping grid (mean inter‐camera distance of 1.31 km, SD = 0.14 km). Due to logistical constrains, the PR and the GR were surveyed in 2017 and the PA was surveyed in 2018. We deployed cameras proportionally to the size of the study areas, with a total of 100 camera‐trap sites in both the PA and the PR and 50 in the GR. Cameras were set for, on average, 75 ± 15 trap‐days (Appendix A: Table A1). At each site, we placed a single, unbaited white‐flash camera (Cuddeback Professional model), 30 cm above ground, 2–3 m away from, and at an angle to, the target animal passage zone, and programmed to photograph at minimum delay (1 s for daytime and 30 s for night‐time). We used the R package camtrapR (Niedballa et al., 2016) to generate daily species‐specific detection histories. A comprehensive description of data collection is provided in Curveira‐Santos, Sutherland, Santos‐Reis, and Swanepoel (2021a).

The target assemblage comprised the 13 wild carnivore species detected across the three study areas (13 in PA, 11 in PR, eight in GR; Curveira‐Santos, Sutherland, Santos‐Reis, & Swanepoel, 2021a), after excluding species with very low occupancy and detection rates unsuitable for detailed analyses (Striped polecat [*Ictonyx striatus*] and Marsh mongoose [*Atilax paludinosus*] in GR) (Appendix A: Table A2).

### 2.2 Spatial co‐occurrence

To estimate pairwise correlations in occupancy among species, we applied a joint species distribution model (JSDMs) that accounts for imperfect detection (Tobler et al., 2019). The JSDM extends multispecies (or community) occupancy models (Dorazio & Royle, 2005) to include residual correlation in species occupancy probabilities via latent variables (Hui et al., 2015). In this model formulation, the true occupancy state of species *i* at site *j* (camera station) is a discrete latent variable *z*_*ij*_. Sampling occurs over *k* occasions (camera days), and the observations *y*_*ijk*_ follow a binomial distribution governed by the probability of detection *p*_*ijk*_. The occupancy component of the model uses a probit regression, with *z*_*ij*_ being governed by a continuous normally distributed latent variable *u*_*ij*_ such that $z_{\mathrm{ij}}=I\left(u_{\mathrm{ij}}>0\right)$. Within the indicator function, *I*(.), the variance of *u*_*ij*_ is constrained by covariate effects and by a set of *T* latent variables $l_{j}=\left(l_{j1},\ldots ,l_{\mathrm{jT}}\right)$ and corresponding species‐specific latent variable coefficients $\theta_{i}=\left(\theta_{i1},\ldots ,\theta_{\mathrm{iT}}\right)$. The latent variables are specified as random variables from a standard normal distribution and represent unmeasured site‐level variation that is attributable to species spatial dependencies. The variance of the residuals ε_ij_ accounts for the variance absorbed by the latent variables and is derived from adjusted variance σi^2^ values for each species *i*. For *T* latent variables and *n* species, the full species correlation matrix R is derived from the correlation in the latent variables as $R=\theta \;\theta^{T}+\mathrm{diag}\left(\sigma_{1}^{2},\sigma_{2}^{2},\ldots ,\sigma_{n}^{2}\right)$.

We were interested in the residual correlation in the occupancy probability that cannot be explained by the environmental covariates in the model, that is, after accounting for species‐specific baseline environmental preferences. Therefore, we modeled species‐specific site occupancy probability as a function of covariates shown to influence species' occupancy patterns in this dataset (see Curveira‐Santos, Sutherland, Santos‐Reis, & Swanepoel, 2021a for details), namely remote‐sensed tree cover estimates within a 500 m radius buffer around each camera station (MODIS vegetation continuous fields; DiMiceli et al., 2011) as a measure of the spectrum of vegetation structure ranging from open grasslands to woodland savannas (TREE). We also considered site‐level (camera) covariates influencing variation in detection probability; specifically, the average width of the trail structure targeted (TRAIL_W), and mean enhanced vegetation index values (MODIS EVI datasets: https://lpdaac.usgs.gov/) for the survey period as a proxy the vegetation density (VEG_D) in the immediate vicinity (30 m) of each site. Prior to analysis, we standardized all area‐specific covariates to have a mean of 0 and standard deviation of 1.

In order to parse out context‐specific residual occupancy correlation patterns, we fitted a single global JSDM for each target area, with the following shared formulation:

(i) Occupancy

$$Z_{\mathrm{ij}}=I\left(u_{\mathrm{ij}}>0\right)$$

$$u_{\mathrm{ij}}=\beta_{0,i}+\beta_{1,i}{\text{TREE}}_{j}+l_{j}\theta_{j}+\varepsilon_{\mathrm{ij}}$$

$$\sigma_{i}^{2}=1-\sum_{t=1}^{T}\theta_{\mathrm{it}}^{2}$$

$$\varepsilon_{\mathrm{ij}}∼\text{Normal}\left(0,\sigma_{i}^{2}\right)$$

(ii) Detection

$$y_{\mathrm{ij}}∼\text{Binomial}\left(k_{j},z_{\mathrm{ij}}\;x\;p_{\mathrm{ij}}\right)$$

$$\text{logit}\left(p_{i,j}\right)=\gamma_{0,i}+\gamma_{1,i}\text{TRAIL}\_W_{j}+\gamma_{2,i}\mathrm{VEG}\_D_{j}$$

The species‐specific regression coefficients $\beta_{1:2,i}$ and $\gamma_{1:2,i}$ (say ω) are treated as species‐specific random effects from a community‐level distribution:

$$\omega_{i}\sim \text{Normal}\left(\mu_{\omega },\sigma_{\omega }\right)$$

We fitted the JSDMs with 7, 6, and 5 latent variables for PA, PR, and GR, respectively, corresponding to about *n*/2 latent variables necessary to approximate the residual correlation matrix when there are *n* species in a community (Tobler et al., 2019). We implemented our models in the BUGS language using the JAGS software (Plummer, 2003) through R. For each co‐occurrence model, we generated three MCMC chains with 30,000 iterations after a 10,000 iteration burn‐in and thinned by 10. We assessed model convergence by a visual inspection of chain trace plots and using the Brooks–Gelman–Rubin statistic (Gelman et al., 2013).

### 2.3 Temporal co‐occurrence

We estimated area‐specific activity patterns for all focal species and calculated pairwise conditional temporal overlap coefficients (i.e., the degree of co‐occurrence in diel activity), using non‐parametric, circular kernel density functions in R package “*circular*” (Oliveira‐Santos et al., 2013). This approach regards species records as random samples of a probability density function representing species activity across a 24‐h period (Ridout & Linkie, 2009). We considered only independent records, defined as those that occurred >1 h after the last detection of the same species, converted to solar time to facilitate ecological interpretation. To describe the general activity period of each species, we used the function “modal.region.circular” to calculate the 95% activity isopleth. We used the function “getBandWidth” to calculate the best smoothing parameter (κ) for each species and maintained the highest value when comparing activity patterns. Finally, using the function “totalvariation,” we calculated pairwise conditional activity overlap coefficients as a measure of temporal co‐occurrence; ranging from 0, for perfect activity dissimilarity, to 1, for full intersection of activity periods (95% isopleths).

### 2.4 Drivers of co‐occurrence

To investigate drivers of spatial and temporal co‐occurrence and how these vary across management contexts, we modeled our co‐occurrence estimates as a function of the species‐pair traits. For spatial co‐occurrence patterns, we fit generalized linear models with the pairwise residual occupancy correlation as the response variable assuming Gaussian errors and an identity link to accommodate the [−1,1] distribution of this response variable. For temporal co‐occurrence, we implemented beta regression models with the temporal activity overlap coefficient as the response variable given the [0,1] distribution of these estimates (Douma & Weedon, 2019). For each response, we created an a priori global model based on the additive effect of the dominance hierarchy within the carnivore guild (i.e., proxy for top‐down pressure) and pairwise measures of ecological similarity. To formally identify and describe how the nature and strength of each covariate effect varies across management contexts, we modeled all covariate effects as interacting with the three‐level “area” covariate (i.e., PA, PR, and GR).

Dominance hierarchy covariates included the different combinations of species' size‐based hierarchy ranks (e.g., “Apex|Large,” and “Large|Medium”) coded as binary variables (Appendix B: Table B1). Lions were ranked as the apex predator, non‐apex species >20 kg as large carnivores, species 5–20 kg as medium‐sized carnivores, and species <5 kg as small carnivores. We also considered models representing the effects of the apex carnivore and other large carnivores on remaining carnivore species, irrespective of size rank, to account for broad suppressive effects over subordinate species of variable sizes. Because of the large number of dominance rank combinations, and hence candidate covariates, we conducted a preliminary analysis to identify the best dominance hierarchy structure for each response (see Appendix C). Only dominance hierarchy covariates selected at this first stage (“Apex|Small” pairings for spatial co‐occurrence and “Large|Medium” pairings for temporal co‐occurrence) were retained in the global models for the drivers of carnivore co‐occurrence.

In addition to dominance hierarchy, we tested the effect of ecological similarity (ES) to explain spatial and temporal co‐occurrence. These ES covariates included diet overlap between pairs of species (calculated as the number of shared food resource categories divided by the total number of unique food categories used by the pair; from Caro & Stoner, 2003), whether a pair included two species of the same hierarchy rank (proxy for lateral competition), and whether a pair included species from the same family (phylogeny effect, proxy for relatedness). Additionally, we modeled the effect of the body‐mass ratio of pairs (including a quadratic relationship) to represent the suggested prevalence of agonistic carnivore interactions at intermediate body‐size differences (body‐mass ratios between 2 and 5.4) when the species are similar enough in size to compete for same prey but different enough so that the larger size of the aggressor entails a low risk of injury (Donadio & Buskirk, 2006). This variable was highly correlated with diet overlap (*r* = 0.70), and to prevent collinearity issues (Dormann et al., 2013), we retained only the body‐mass ratio in the global models since it led to an higher *R* ^2^ for both the spatial and temporal dimensions. Finally, we considered the effects of the species spatial co‐occurrence on temporal overlap and temporal overlap on spatial co‐occurrence to evaluate complementarity in niche partitioning patterns (Schoener, 1974).

## 3 RESULTS

**FIGURE 2:**
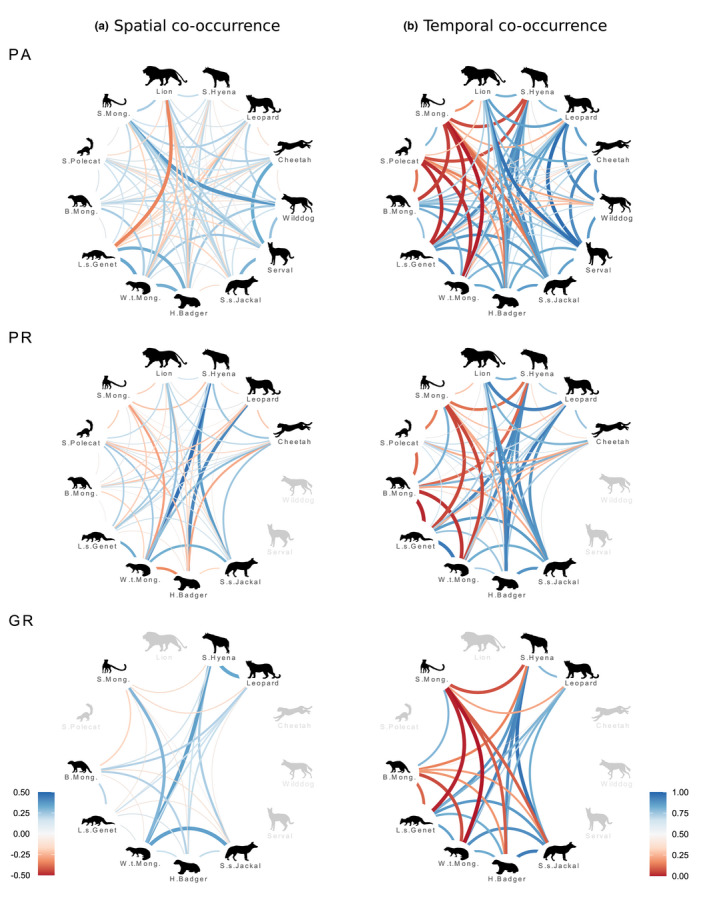
Schematic depictions of pairwise (a) spatial and (b) temporal co‐occurrence estimates within South African carnivore assemblages, across three adjacent management contexts (PA—provincial protected area; PR—private game reserve; GR—commercial game ranches). Body size in the carnivore assemblage decreases clockwise. Spatial co‐occurrence patterns were estimated as pairwise residual occupancy correlation values from a hierarchical Bayesian joint species distribution model, and temporal co‐occurrence expressed as pairwise coefficients of diel activity overlap from non‐parametric, circular kernel density functions (see Methods). Line widths are proportional to the estimated co‐occurrence values.

Our dataset included a total of 4895 independent records of our 13 target wild carnivore species over 19,739 effective trap‐days (Appendix A: Table A2). We estimated spatial (residual correlation in occupancy probability) and temporal (diel activity overlap) co‐occurrence for a total of 161 species pairs: 78 in PA, 55 in PR, and 28 in GR (Figure 2).

### 3.1 Spatial co‐occurrence

**FIGURE 3:**
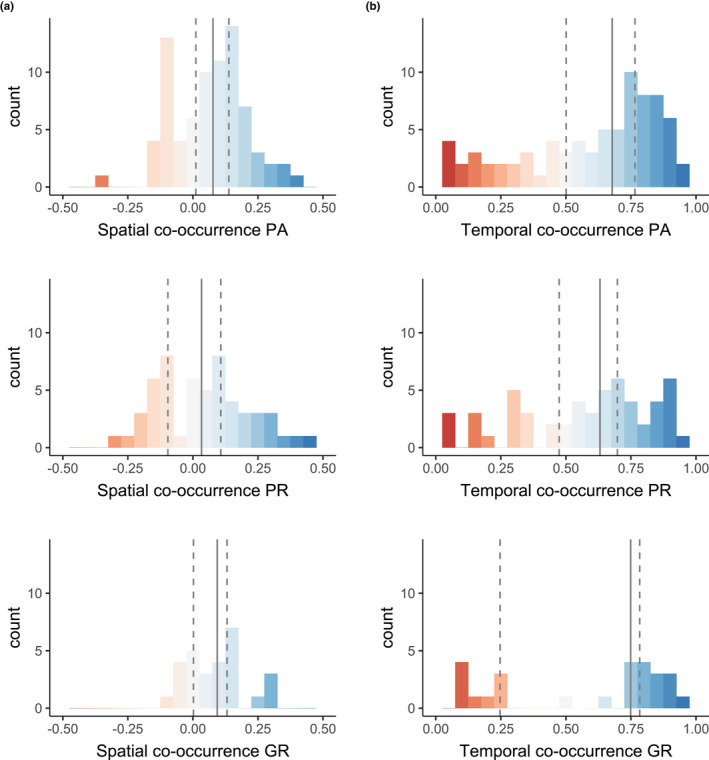
Assemblage‐level (a) spatial (residual occupancy correlation) and (b) temporal (diel activity overlap) carnivore co‐occurrence patterns in each of the three management contexts (PA—provincial protected area; PR—private game reserve; GR—commercial game ranches). Vertical lines mark area‐specific medians (solid line) and 33 and 66% quantiles (dashed lines).

**FIGURE C1:**
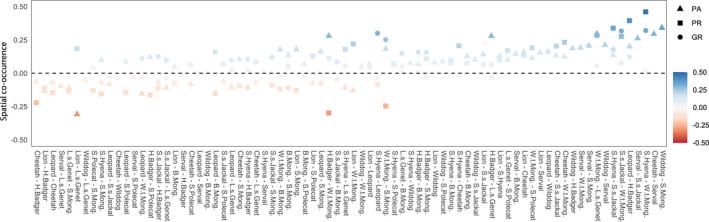
Spatial co‐occurrence patterns across species‐pair and area combinations. PA, provincial protected area; PR, private game reserve; GR, commercial game ranches. Pairwise estimates are residual occupancy correlation values from a hierarchical Bayesian joint species distribution model (see Methods). Credible intervals were omitted for visual clarity. Pairs are ordered by average co‐occurrence values across the three areas.

The mean residual correlation in occupancy across all species pair and area combinations was 0.06 ± 0.15 (mean ± SD), indicating that, at the assemblage level, there was neutral or independent spatial co‐occurrence. Area‐specific means exhibited a similar pattern (PA = 0.06 ± 0.12, PR = 0.03 ± 0.16, and GR = 0.08 ± 0.11), albeit with large variation across species pairs (Figure 3a). Pairwise residual occupancy correlation values ranged from 0.46 (95% Bayesian credible intervals, 0.27–0.64), between the spotted hyaena and the white‐tailed mongoose (*Ichneumia albicauda*) in the private reserve, to −0.36 (−0.52 to −0.05), between the lion and the large‐spotted genet (*Genetta maculata*) also in the private reserve (Figure 2a; Appendix C: Figure C1). Positive spatial dependencies were predominant in both the protected area (67%) and the game ranches (68%), while positive and negative residual correlation values were more evenly distributed in the private reserve (56% vs. 44%, respectively, Figure 3a). However, strong evidence for non‐independent spatial co‐occurrence patterns (>0.9 probability of a different than zero correlation) was only observed in 14 out of 161 pair‐by‐area combinations; 12 of which were positive (five in PA, five in PR, and three in GR) and only two were negative (one in PA and PR each). Notably, the strength of negative spatial co‐occurrence signals in game ranches was very low (>−0.1), with no pairs exhibiting a >0.7 probability of a spatial avoidance pattern.

### 3.2 Temporal co‐occurrence

**FIGURE C2:**
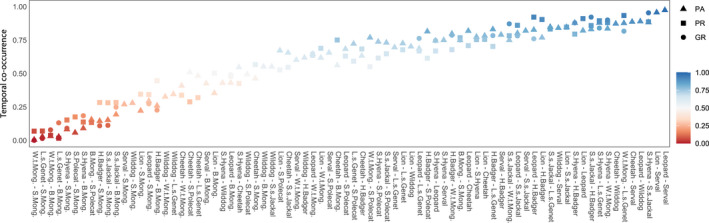
Temporal co‐occurrence patterns across species‐pair and area combinations. PA, provincial protected area; PR, private game reserve; GR, commercial game ranches. Pairwise estimates are coefficients of diel activity overlap from non‐para‐metric, circular kernel density functions (see Methods). Pairs are ordered by average co‐occurrence values across the three areas.

Pairwise temporal activity overlap coefficients spanned a wide range of values (Figure 2b; Appendix C: Figure C2), from no temporal co‐occurrence between strictly nocturnal and diurnal species (0.00 between slender [*Herpestes sanguineus*] and white‐tailed mongooses in both the PA and GR) to nearly complete activity overlap (0.97 between leopard and serval [*Leptailurus serval*] in PA), thus indicating an intricate temporal co‐occurrence structure at the assemblage level (Figure 3b). Nonetheless, moderate‐to‐high overlap values (>0.5) were common in all three areas (69% in PA, 65% in PR, and 57% in GR) due to a high number of predominantly nocturnal species in all the assemblages. While the distribution of activity overlap values was similar between the protected area and the private reserve, the pattern in the game ranches was bimodal, that is, either low or high overlap. This was mostly attributable to the absence of several species with cathemeral activity patterns (e.g., lion and serval) rather than accentuated species‐specific changes in activity (see below).

### 3.3 Context‐dependency in co‐occurrence patterns

**FIGURE 4:**
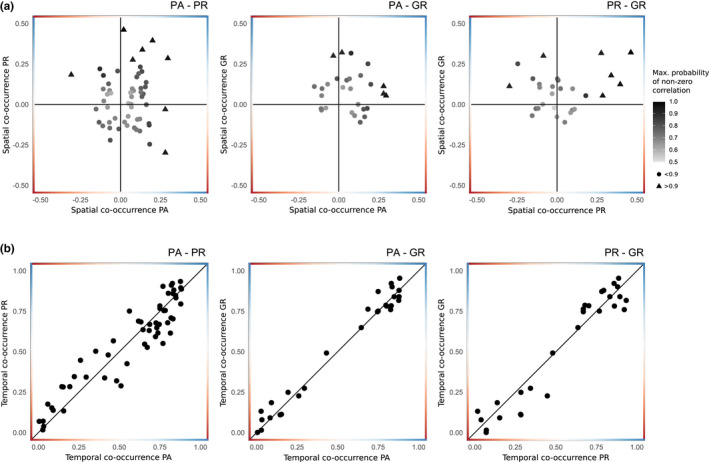
Context‐dependency in (a) spatial (residual occupancy correlation) and (b) temporal (diel activity overlap) co‐occurrence patterns for species pairs present in two or more areas (*n* = 55). PA, provincial protected area; PR, private game reserve; GR, commercial game ranches. For the spatial dimension, points in the top‐right and bottom‐left quadrants indicate a consistent co‐occurrence signal, while top‐left and bottom‐right quadrants indicate context‐dependent spatial dependencies. The color and shape of the points represents the proportion of the posterior distribution of the residual occupancy estimate with the same sign as the mean, that is, the probability for a different than zero correlation. For the temporal dimension, consistency in diel activity overlapped is expressed by proximity to the diagonal bar. Credible and confidence intervals were omitted for visual clarity.

Considering only species pairs present in more than one area (*n* = 55), context‐dependency in pairwise spatial dependencies was frequent whereas temporal co‐occurrence patterns were largely consistent across areas (Figures 2 and 4; Appendix C: Figures C1 and C2). Although weak residual occupancy correlation strength was the norm, nearly half (55%) of species pairs exhibited contrasting signals in residual occupancy correlations across areas, that is, context‐dependency. Species with high probability of spatial avoidance in one context (e.g., lion and large‐spotted genet in PA), specifically, rarely did so in the others. Consistency in positive spatial dependencies across contexts was more common; however, seldom pairs exhibited correlation values that strongly departed from a hypothesis of independence (>0.9 probability) in different contexts. Conversely, species' temporal co‐occurrence patterns were similar between contexts, with 75% of pair‐specific diel activity overlap differences of ≤0.1 across areas.

### 3.4 Drivers of co‐occurrence

The main drivers of carnivore spatial co‐occurrence differed among management contexts (Table 1a). In the protected area, “Apex|Small” dominance hierarchy pairs, that is, pairs with the lion and a small‐sized carnivore, exhibited lower spatial co‐occurrence relative to all other pairs. In the private reserve, spatial co‐occurrence was lower for pairs from the same taxonomic family, but increased for pairs with higher diel activity overlap (i.e., pairs with high temporal overlap also exhibited high spatial overlap) and of intermediate body‐mass differences (ratios ~2), that is, the residual occupancy correlation varied by a concave quadratic effect of the pairs' body‐mass ratio. No clear associations were found to explain variability in species' spatial co‐occurrence in the game ranches.

**TABLE 1:** Beta coefficient estimates (±SE) for species‐pair‐specific covariates representing the hypothesized drivers of carnivore (a) spatial and (b) temporal co‐occurrence

Covariate		(a) Spatial co‐occurrence			(b) Temporal co‐occurrence		
		PA	PR	GR	PA	PR	GR
Dominance hierarchy	Apex|Small	**−0.18 ± 0.08**	0.13 ± 0.08	‐	‐	‐	‐
	Large|Medium	‐	‐	‐	0.58 ± 0.34	0.54 ± 0.46	0.98 ± 0.57
Same rank	Large	−0.07 ± 0.06	0.14 ± 0.09	0.21 ± 0.16	0.17 ± 0.46	0.20 ± 0.65	−0.91 ± 1.23
	Medium	−0.09 ± 0.10	−0.08 ± 0.16	0.02 ± 0.17	0.43 ± 0.74	1.23 ± 1.14	0.13 ± 1.27
	Small	−0.02 ± 0.06	0.12 ± 0.07	−0.02 ± 0.11	**−1.73 ± 0.42**	**−1.15 ± 0.50**	**−1.98 ± 0.75**
Same family		0.07 ± 0.04	**−0.18 ± 0.06**	−0.02 ± 0.11	0.46 ± 0.33	0.30 ± 0.47	−0.27 ± 0.84
Body‐mass ratio		−0.10 ± 0.05	**0.14 ± 0.06**	0.03 ± 0.10	−0.15 ± 0.37	0.11 ± 0.45	−0.78 ± 0.71
Body‐mass ratio^2^		0.02 ± 0.01	**−0.03 ± 0.01**	−0.01 ± 0.02	−0.01 ± 0.06	−0.04 ± 0.07	0.07 ± 0.13
Temporal co‐occurrence		−0.08 ± 0.06	**0.22 ± 0.08**	0.06 ± 0.09	‐	‐	‐
Spatial co‐occurrence		‐	‐	‐	−1.04 ± 0.89	**2.10 ± 0.84**	1.70 ± 1.71
**Global model *R*** ^**2**^		0.25			0.39		

*Note*: Well‐supported coefficients (*p* < .05) are bolded.

Abbreviations: PA, provincial protected area; PR, private game reserve; GR, commercial game ranches.

The global model for the drivers of carnivore temporal co‐occurrence (Table 1b) indicated that, in all areas, pairings that included two small‐sized species exhibited less temporal overlap than other size pairings. Additionally, only in the private reserve, species with high spatial overlap also exhibited high temporal activity overlap.

## 4 DISCUSSION

Our study provides important insights into context‐dependency of carnivore spatial and temporal co‐occurrence across a South African multi‐tenure conservation landscape. We found that carnivores generally distribute independently across space. Clear pairwise spatial dependencies were rare in all areas and seldom consistent across management and conservation models. Yet, as hypothesized, a higher overall degree of spatial overlap was observed in the protected area, where species generally occur at higher relative abundances, and in game ranches, where human disturbance presumably narrows the scope for spatial asymmetries. In the private reserve, spatial co‐occurrence patterns were more heterogeneous but did not support the hypothesis of a well‐marked dominance hierarchy associated with higher apex predator densities. In contrast, species‐pairs diel activity overlap was widespread and stable across areas, suggesting that, in our study areas, temporal asynchrony is less affected by the local context but regulated by each species' endogenous clock and adaptations to long‐term coexistence with dominant predators. Our results suggest that carnivore interspecific interactions may have limited expression in species co‐occurrence patterns, but existing spatial dependencies may reflect varying outcomes of context‐specific interactions.

Irrespective of the context, the tendency for weak residual spatial dependencies among species pairs indicates that carnivores were generally distributed independently over the landscape. But note that partitioning data by area for insights on context‐dependency likely promotes the underestimation of co‐occurrence strength (Tobler et al., 2019). Among observable spatial dependencies, we found that carnivore species were more likely to overlap spatially rather than avoid each other. Such observations corroborate global‐scale patterns of carnivore co‐occurrence described by Davis et al. (2018) and the general assertion that the relative distribution of sympatric carnivore species is mostly driven by species‐specific preferences and resource use affinities. Other studies have found that sympatric carnivores with similar ecological traits predominantly select for the same sites (Davis et al., 2018; Jonathan Davies et al., 2007; Monterroso et al., 2020). In our assessment of spatial dependencies, we attempted to account for environmental filtering using a broad proxy of habitat structure (i.e., tree cover). However, this likely fails to fully capture other, more fine‐scale and resource‐relevant habitat selection factors, such as prey availability, which may underly local scale intraguild sympatry and heterogeneity of the competitive environment (Amarasekare, 2003). Moreover, under a common stressor, species may aggregate in spatial refugia (Farris et al., 2017; Sogbohossou et al., 2018). In the game ranches, where larger and putative problem species (hyaena and leopard) experience persecution (Balme et al., 2010; Pitman et al., 2017), these carnivores overlapped spatially. In such areas, the influence of human disturbance on these species may incur fitness costs through increased competition in shared, low‐risk sites (Sévêque et al., 2020). While the reduced level of spatial asymmetries seemingly contradicts the “competitive exclusion” and “limiting similarity” principles (Macarthur & Levins, 1967), our coarse‐scale analysis of spatial dependencies precluded exploration of fine‐scale and spatiotemporally explicit avoidance and resource partitioning mechanisms. Many subordinate carnivores minimize the risk of encountering dominant competitors in resource‐rich sites by fine‐scale avoidance behaviors and constrained predation strategies (Ramesh et al., 2017).

Despite the predominance of independent or overlapping spatial patterns, previous studies have suggested that, albeit rare, spatial avoidance is most common for pairs including large‐bodied carnivores (Davis et al., 2018), able to exert strong suppression over subordinate species and induce avoidance behaviors (Donadio & Buskirk, 2006; Ritchie & Johnson, 2009). We were particularly interested in how the spatial structuring of the competitive environment may uphold coexistence between competition‐depressed mesopredators and the densely distributed apex predator population in the private reserve. The few depressed residual occupancy correlation values observed included variable body‐size pairings and dominance hierarchies. However, particularly in the protected area, small carnivore species tended to spatially avoid lions. This is in line the with recently uncovered susceptibility of small African carnivores to lion suppression (Curveira‐Santos et al., 2022; Curveira‐Santos, Sutherland, Tenan, et al., 2021b). Unlike larger subordinate species, better equipped to escape and mitigate dangerous encounters while co‐occurring with the apex predator, for small carnivores spatial avoidance of lions may be a better strategy (Wirsing et al., 2010). Collectively, however, our results reinforce the notion that information on species spatial co‐occurrence, while useful to characterize the setting of potential ecological interactions, may be a poor proxy for the actual signal and strength of interactions (Blanchet et al., 2020). Detailed behavioral investigations and, importantly, an increased understanding of carnivore interactions on population demography remain central to unraveling potential suppression patterns (Miller et al., 2018).

Temporal partitioning is acknowledged as an important structuring force in carnivore assemblages, facilitating species coexistence (Di Bitetti et al., 2010; Hayward & Slotow, 2009), particularly with increasing assemblage complexity (Monterroso et al., 2014). In our multi‐carnivore system, this was evidenced by the full range of pairwise activity overlap values observed. Diel activity asynchrony was well‐marked between the diurnal species (slender and banded [*Mungos mungo*] mongooses) and remaining carnivores with reduced or partial daytime activity (most large‐ and medium‐sized species), especially sympatric smaller carnivores with predominant nocturnal habits (genet, white‐tailed mongoose and stripped polecat). This corroborates the general understanding of these species' ecology (Hunter, 2018). Among larger species, varying degrees of diurnal and crepuscular activity, coupled with frequent asynchronous activity peaks, often resulted in moderate overlap values. Staple prey of large African carnivores are available throughout the day in African savanna ecosystems, and competition avoidance has been proposed as the primary cause of temporal partitioning among these carnivore species (Hayward & Slotow, 2009).

Remarkably, we found that temporal overlap remained similar across contexts, despite changes in guild composition, species relative abundance, and other extrinsic factors. Previous studies have suggested plasticity in circadian activity patterns of carnivores may enable coexistence across different ecological contexts (Monterroso et al., 2014). However, our results suggest that, in our study areas, interspecific temporal partitioning among carnivores may be maintained within species' own endogenous boundaries, which have evolved under long‐term coexistence with dominant sympatric predators. Importantly, low human encroachment in all our focal areas likely does not translate into enough pressure to induce increased nocturnality, as observed in other systems (Gaynor et al., 2018). It is possible, however, that our area‐level measure of activity overlap fails to capture micro‐adjustments in activity peaks across contexts.

Interspecific interactions are multidimensional. Niche partitioning theory suggests that in order to reduce competitive stress and facilitate coexistence, species converging along one niche axis, here space or time, segregate in complementary dimensions (Schoener, 1974). However, in both the protected area and game ranches, we found that species dependencies across the two studied dimensions were largely unrelated. Notably, in the private reserve, co‐occurrence in space and time was the main underlying assemblage structure. Again, if environmental and resource affinities, and not intraguild interactions and competition, are the primary driver of carnivore co‐occurrence, such patterns may simply translate to common strategies by which groups of sympatric carnivores exploit their environment in each context. Such reasoning reinforces the need to consider the potential role of fine‐scale behavioral adjustments for a sound understanding of the mechanisms regulating co‐occurrence patterns (Cusack et al., 2016; Vanak et al., 2013). Importantly, such spatial and temporal relationships are theoretically underpinned by the degree of trophic niche sharing (the third and missing fundamental niche axis, Schoener, 1974) and varying prey preferences and predatory strategies (Hayward & Kerley, 2008).

Prevalent patterns of independency in species spatial distribution and consistent temporal activity overlap across areas did not fully support the hypothesized relationship between the local management context and assemblage‐level signals in species co‐occurrence. However, we observed that well‐marked pairwise spatial dependencies were idiosyncratic, with same species occurring independently or with opposing signal in one of the adjacent landscapes. Accordingly, the role of carnivore dominance relationships and ecological similarity in mediating co‐occurrence patterns was context‐specific. Such pair‐by‐area variability suggests that subordinate carnivores may alternate between pre‐emptive behavioral strategies to avoid dominant competitors and instances of fine‐scale co‐occurrence (Karanth et al., 2017). The latter may or may not be accompanied by strategies to mitigate competitive stress at shared sites, such as increased vigilance or facultative character displacement (e.g., prey switching; Pfennig et al., 2006). The resulting co‐occurrence pattern and, importantly, the fitness impacts of species co‐occurrence in a given context, likely depends on the complex interplay of density‐ and trait‐mediated (e.g., phenotypic plasticity and body‐size asymmetry) effects and characteristics of the local environment (e.g., landscape features, resource availability and diversity, and disturbance; Werner & Peacor, 2003).

By closely inspecting changes in co‐occurrence of species pairs with spatial dependencies of varying signal across areas, we observed that spatial overlap between the same species was generally more common in both the protected area and game ranches, and spatial segregation more frequent in the private reserve. Compared with the private reserve, the scope for pre‐emptive spatial avoidance may be reduced because of widespread distribution of dominant species in the protected area or limited low‐risk spaces in the game ranches. Detailed behavioral and demographic research is needed to ascertain whether potentially increased carnivore encounter rates may enhance competitive and suppression pathways or if spatial avoidance in the private reserve is itself the outcome of amplified competitive stress. Nevertheless, our results suggest that understanding nonstationary properties of carnivore interactions may be important to avoid erroneous extrapolation to local policies and practices (Rollinson et al., 2021).

With our study, we provide novel insights into the potential of alternative management and conservation models to influence community‐wide ecological patterns and processes, specifically, the context‐dependency of spatial and temporal associations of African carnivore species. Our empirical exploration of co‐occurrence patterns emphasizes three important aspects underlying carnivore community research: the value of multispecies assessments (Heim et al., 2019), the importance of predator behavior and interspecific interactions (Ritchie & Johnson, 2009), and the prevalence of context‐dependency in ecological interactions (Chamberlain et al., 2014). Understanding such aspects and how they relate to management interventions, particularly under southern Africa's decentralized conservation approaches and predator‐oriented management paradigms (Caro, 2015; Pitman et al., 2017), is of interest to carnivore conservation efforts and for managing and protecting food webs (Estes et al., 2011; Ritchie et al., 2012). However, our research also exemplifies the challenge of studying multiple species and environmental contexts, when resulting patterns are the likely outcome of a complex web of spatially structured intraguild relationships that may mask individual‐species associations, themselves ruled by inconspicuous idiocracies of species behavior.

## AUTHOR CONTRIBUTIONS

**Gonçalo Curveira‐Santos** involved in conceptualization (lead); data curation (lead); formal analysis (lead); funding acquisition (supporting); investigation (lead); methodology (lead); project administration (equal); visualization (lead); writing—original draft (lead); and writing—review and editing (lead). **Laura Gigliotti:** involved in conceptualization (supporting); formal analysis (supporting); investigation (supporting); methodology (supporting); writing—original draft (supporting); and writing—review and editing (equal). **Chris Sutherland** involved in conceptualization (supporting); formal analysis (supporting); investigation (supporting); methodology (supporting); supervision (equal); and writing—review and editing (equal). **Daniela Rato** involved in conceptualization (supporting); formal analysis (supporting); investigation (supporting); methodology (supporting); and writing—review and editing (supporting). **Margarida Santos‐Reis** involved in conceptualization (supporting); funding acquisition (supporting); methodology (supporting); supervision (equal); and writing—review and editing (equal). **Lourens H. Swanepoel** involved in conceptualization (supporting); data curation (supporting); formal analysis (supporting); funding acquisition (lead); investigation (supporting); methodology (supporting); project administration (lead); supervision (equal); and writing—review and editing (equal).

## CONFLICT OF INTEREST

The authors have no competing interests or conflict of interest to declare.

## Data Availability

Data are available via the figshare repository https://doi.org/10.6084/m9.figshare.16965340.v1.

## APPENDIX A

### Camera‐trapping survey information and summary of carnivore detection data and occupancy estimates

**TABLE A1:** Summary of camera‐trapping surveys across three adjacent wildlife‐oriented management contexts in the Maputaland region of northern KwaZulu‐Natal, South Africa

Area (management context)	Survey year	Camera‐traps	Effort (camera‐days)
Provincial protected area (uMkhuze section of iSimangaliso Wetland Park)	2018	100	6925
Private ecotourism reserve (Mun‐ya‐wana Private Game Reserve)	2017	100	8578
Commercial game ranches	2017	50	4236
	Total	250	19,739

**TABLE A2:** Number of independent photographic detections (<1 h) and species‐area‐specific mean realized occupancy probability estimates from the joint species distribution model, for the 13 target carnivore species recorded in camera‐trapping surveys across the three management contexts

Species	Latin name	Family	Bodymass (kg)	Detections			Mean occupancy		
				PA	PR	GR	PA	PR	GR
African lion	*Panthera leo*	Felidae	176	38	107	‐	0.28	0.61	‐
Spotted hyaena	*Crocuta crocuta*	Hyaenidae	63	485	125	23	0.90	0.62	0.46
Leopard	*Panthera pardus*	Felidae	53.8	408	154	107	0.89	0.46	0.63
Cheetah	*Acinonyx jubatus*	Felidae	46.5	21	33	‐	0.39	0.60	‐
African wild dog	*Lycaon pictus*	Canidae	26.5	34	‐	‐	0.21	‐	‐
Serval	*Leptailurus serval*	Felidae	13.4	16	‐	‐	0.17	‐	‐
Side‐striped Jackal	*Canis adustus*	Canidae	10.3	68	95	10	0.25	0.40	0.25
Honey badger	*Mellivora capensis*	Mustelidae	10	83	33	8	0.50	0.41	0.50
White‐tailed mongoose	*Ichneumia albicauda*	Herpestidae	3.5	577	169	63	0.82	0.60	0.61
Large‐spotted genet	*Genetta maculata*	Viverridae	2	1176	481	365	0.93	0.80	0.74
Banded mongoose	*Mungus mungus*	Herpestidae	1.6	58	18	32	0.51	0.31	0.49
Striped polecat	*Ictonyx striatus*	Mustelidae	0.9	5	13	‐	0.08	0.48	‐
Slender mongoose	*Herpestes sanguineus*	Herpestidae	0.6	64	12	14	0.48	0.63	0.41

*Note*: PA, provincial protected area; PR, private game reserve; GR, commercial game ranches.

## APPENDIX B

### Preliminary dominance hierarchy models

We conducted a preliminary analysis to determine the best dominance hierarchy covariates to be included in the global models of carnivore co‐occurrence drivers. We ran single covariate models corresponding to the different dominance hierarchy combinations (Table B1) for each co‐occurrence dimension (generalized linear model for the spatial models and beta regression models for the temporal models, see Methods for details) and management context separately. We ranked models based on Akaike's Information Criterion corrected for sample size (AICc), and retained all the dominance hierarchy covariates in models within ΔAICc ≤ 2 (Table B2). If the null model was also ΔAICc ≤ 2, we did not retain covariates from any of the competitive models.

**TABLE B1:** Candidate models for determining the dominance hierarchical rank structure of carnivore spatial and temporal co‐occurrence. Lions were ranked as the apex predator, non‐apex species >20 kg as large carnivores, species 5–20 kg as medium‐sized carnivores, and species <5 kg as small carnivores

Candidate model	Covariate
Apex|All	Pairings with the apex carnivore and any other species, irrespective of rank (1) vs. all other pairings (0)
Large|All	Pairings with the apex or a large carnivore and any other species, irrespective of rank (1) vs. all other pairings (0)
Apex|Large	Pairings with the apex and a large‐sized carnivore (1) vs. all other pairings (0)
Apex|Medium	Pairings with the apex and a medium‐sized carnivore (1) vs. all other pairings (0)
Apex|Small	Pairings with the apex and a small‐sized carnivore (1) vs. all other pairings (0)
Large|Medium	Pairings with a large‐ and a medium‐sized carnivore (1) vs. all other pairings (0)
Large|Small	Pairings with a large‐ and a small‐sized carnivore (1) vs. all other pairings (0)
Medium|Small	Pairings with a medium‐ and a small‐sized carnivore (1) vs. all other pairings (0)

**TABLE B2:** Model selection results for dominance hierarchy structure models. Retained covariates are indicated in bold

Model	k^a^	Protected area (PA)		Private reserve (PR)		Game ranches (GR)	
		ΔAICc	w^b^	ΔAICc	w^b^	ΔAICc	w^b^
Spatial co‐occurrence							
**Apex|Small**	3	**0**	**0.47**	2.02	0.08	‐	‐
Large|All	3	2.74	0.12	1.08	0.13	2.46	0.13
Null	2	3.17	0.10	0	0.23	0	0.46
Apex|All	3	4.00	0.06	1.99	0.08	‐	‐
Medium|Small	3	4.07	0.06	1.87	0.09	2.31	0.14
Large|Small	3	4.10	0.06	2.09	0.08	2.51	0.13
Large|Medium	3	4.36	0.05	1.17	0.13	2.46	0.13
Apex|Large	3	4.76	0.04	1.79	0.09	‐	‐
Apex|Medium	3	5.31	0.03	2.00	0.08	‐	‐
Temporal co‐occurrence							
**Large|Medium**	3	**0**	**0.58**	**0**	**0.30**	**0**	**0.38**
Apex|Medium	3	3.62	0.09	1.71	0.13	‐	‐
Large|All	3	4.42	0.06	2.14	0.10	1.03	0.23
Null	2	4.45	0.06	1.97	0.11	0.81	0.25
Large|Small	3	4.51	0.06	3.94	0.04	3.28	0.07
Apex|All	3	4.66	0.06	1.48	0.14	‐	‐
Apex|Large	3	5.39	0.04	2.04	0.11	‐	‐
Apex|Small	3	6.46	0.02	4.20	0.04	‐	‐
Medium|Small	3	6.48	0.02	4.21	0.04	3.33	0.07

^a^ Number of model parameters.

^b^ Akaike model weight.
